# Mechanism of norcantharidin intervention in gastric cancer: analysis based on antitumor proprietary Chinese medicine database, network pharmacology, and transcriptomics

**DOI:** 10.1186/s13020-024-01000-1

**Published:** 2024-09-17

**Authors:** Yiyan Zhai, Fanqin Zhang, Jiying Zhou, Chuanqi Qiao, Zhengsen Jin, Jingyuan Zhang, Chao Wu, Rui Shi, Jiaqi Huang, Yifei Gao, Siyu Guo, Haojia Wang, Keyan Chai, Xiaomeng Zhang, Tieshan Wang, Xiaoguang Sheng, Xinkui Liu, Jiarui Wu

**Affiliations:** 1 School of Chinese Materia Medica, Beijing University of Chinese Medicine, Beijing, 100029 China; 2 Beijing Research Institute of Chinese Medicine, Beijing University of Chinese Medicine, Beijing, 100029 China; 3 Innovative Institute of Chinese Medicine and Pharmacy, Shandong University of Traditional Chinese Medicine, Jinan, 250355 Shandong China

**Keywords:** Antitumor patent Chinese medicine, Database, Gastric cancer, SERPINE 1

## Abstract

**Background:**

Combining antitumor proprietary Chinese medicine (pCm) with radiotherapy and chemotherapy can effectively improve tumor cure rates and enhance patients’ quality of life. Gastric cancer (GC) severely endangers public health. Despite satisfactory therapeutic effects achieved by using antitumor pCm to treat GC, its underlying mechanism remains unclear.

**Objective:**

To integrate existing research data, construct a database of antitumor pCm, and study the intervention mechanisms in GC by focusing on their monomer components.

**Methods:**

We constructed an antitumor pCm database based on China’s medical insurance catalog, and employed network pharmacology, molecular docking methods, cell experiments, transcriptomics, and bioinformatics to investigate the intervention mechanisms of effective pCm components for GC.

**Results:**

The study built an antitumor pCm database including 55 pCms, 171 Chinese herbal medicines, 1955 chemical components, 2104 targets, and 32 disease information. Network pharmacology and molecular docking technology identified norcantharidin as an effective component of antitumor pCm. In vitro experiments showed that norcantharidin effectively inhibited GC cell proliferation, migration, and invasion; blocked the G2/M cell cycle phase; and induced GC cell apoptosis. Transcriptomic results revealed that norcantharidin affected biological processes, such as cell adhesion, migration, and inflammatory responses by influencing PI3K-AKT, NF-κB, JAK-STAT, TNF-α signaling pathways, and EMT-related pathways. Core molecules of norcantharidin involved in GC intervention include SERPINE1, SHOX2, SOX4, PRDM1, TGFR3, TOX, PAX9, IL2RB, LAG3, and IL15RA. Additionally, the key target SERPINE1 was identified using bioinformatics methods.

**Conclusion:**

Norcantharidin, as an effective component of anti-tumor pCm, exerts its therapeutic effects on GC by influencing biological processes such as cell adhesion, migration, and inflammation. This study provides a foundation and research strategy for the post-marketing re-evaluation of antitumor pCms.

**Supplementary Information:**

The online version contains supplementary material available at 10.1186/s13020-024-01000-1.

## Introduction

As research on the modernization of Traditional Chinese Medicine (TCM) progresses and modern preparation processes and production techniques continue to advance rapidly, there has been a notable increase in the use of antitumor proprietary Chinese medicines (pCms) for the treatment of malignant tumors. For instance, Aidi injection is extensively employed to treat liver cancer, lung cancer, colorectal cancer, and non-small cell lung cancer, among other malignancies [1]. In contrast, compound Kushen injection is prevalently used for non-small cell lung cancer, gastrointestinal tumors, and primary liver cancer [2].

Gastric cancer (GC), a malignant tumor arising from the gastric mucosal epithelial cells, is reported in the *2021 Global Cancer Statistics* as ranking 5th in incidence and 4th in mortality. In China, the number of new GC cases and resulting deaths holds the 3rd position among malignant tumors. Due to the difficulty in diagnosing early-stage GC, most cases are identified during the progressive stage, leading to an overall five-year survival rate of below 50% [3, 4]. Radiotherapy and chemotherapy are commonly employed in GC treatment; however, the significant side effects lead to an unsatisfiable quality of life for patients.

Numerous studies have demonstrated that antitumor pCms can help reduce adverse reactions caused by tumor radiotherapy and chemotherapy while enhancing their efficacy through unique mechanisms [5–8]. For example, Aidi injection can regulate the PI3K-AKT signaling pathway, inhibit ERK1/2 protein phosphorylation levels, upregulate Caspase-3, Caspase-9, Fas, Fasl, and Cyt-c expression levels, suppress NF-κB activation, downregulate Bcl-2 protein expression levels, decrease mitochondrial membrane potential, promote tumor cell apoptosis, inhibit DNA synthesis in S-phase tumor cells, arrest tumor cells in the G2/M phase, and hinder their proliferation [9, 10]. Although the action mechanisms of various antitumor pCms have been widely studied, their specific mechanisms remain unclear, primarily due to the complex chemical components of most compound preparations.

A significant amount of basic research has led to a continuous increase in data related to traditional Chinese medicine (TCM). This has resulted in the creation of numerous and diverse TCM databases, effectively advancing TCM research and development. Commonly used databases include TCMSP, ETCM, and HERB, among others. For instance, ETCM (The Encyclopedia of Traditional Chinese Medicine) provides standardized information on commonly used TCM, TCM prescriptions, and their chemical components. This database offers information on TCM formulas, chemical constituents, and corresponding targets for user retrieval. Additionally, it can predict targets based on the similarity between TCM components and known drug chemical structures, and perform Gene Ontology (GO) function and Kyoto Encyclopedia of Genes and Genomes (KEGG) pathway enrichment analyses [11]. In summary, these databases primarily focus on individual TCM research, encompassing extensive information on TCM components, targets, and diseases, which can be used for TCM target prediction.

Despite the progress made in TCM database research, certain issues persist. Some databases emphasize information retrieval, while others concentrate on the systematic analysis of drug mechanisms. The reliability of data analysis from these databases is often inadequate, and the lack of uniform inclusion and analysis standards remains a challenge for TCM researchers. Developing standardized and normative databases is still an urgent need. Moreover, antitumor pCms listed in the medical insurance catalog are widely used in clinical oncology treatments. These medicines have undergone numerous clinical trials, demonstrating significant efficacy and minimal adverse reactions, thereby ensuring clinical effectiveness and medication safety for patients. Inclusion in the medical insurance catalog also implies a price advantage, further benefiting patients. Thus, research into the mechanisms of action of antitumor pCms included in the medical insurance catalog holds great promise. However, there is currently no database constructed specifically based on the proprietary Chinese medicines listed in the medical insurance catalog.

Network pharmacology and molecular docking techniques are emerging as effective approaches to identify key components of pCms. Network pharmacology allows for the creation of multi-level biological networks, aligning with the “multi-targets, multi-components” nature of traditional Chinese medicine. Molecular docking techniques simulate and predict the binding patterns between drug molecules and their target actions at the computational level [12, 13]. Reliable databases are essential for utilizing network pharmacology and molecular docking, but such databases for antitumor pCms are currently lacking. The rapid progress of high-throughput technologies is accelerating research into the mechanisms of action for various anti-tumor drugs. Transcriptome analyses help to reveal mechanisms and targets from different perspectives; combining these techniques with bioinformatics analysis methods significantly enhance prediction accuracy.

**Fig. 1:**
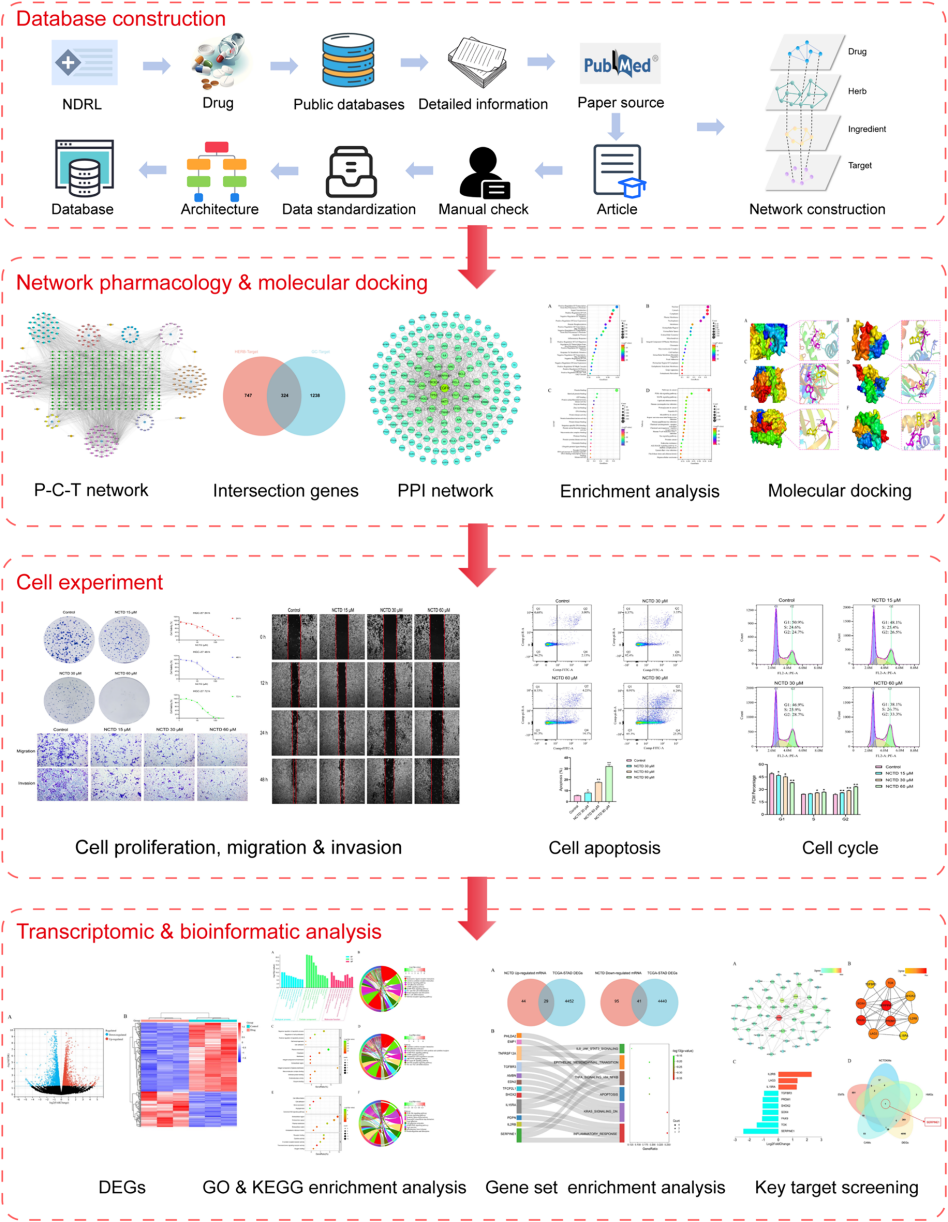
Workflow of this study

This study relies on the constructed antitumor pCm database, using the individual components as the entry point. This approach aims to elucidate the overall mechanisms of action of antitumor pCms, which may prove to be an effective strategy. The workflow of this study is depicted in Fig. 1.

## Materials and methods

### Sources of database information

#### Data of antitumor pCms

Information on antitumor pCms is retrieved from China’s *Catalogue of Drugs for Basic National Medical Insurance/Employment Injury Insurance/Birth Insurance (2022)* (NRDL). The antitumor pCms included in the NRDL are widely applied in clinical treatment. These medicines have undergone multiple clinical trials, demonstrating significant therapeutic effects, minimal side effects, and ensuring clinical efficacy and medication safety for patients. Moreover, inclusion in the NRDL also indicates a pricing advantage, which benefits patients greatly.

#### Data of Chinese materia medica

Information on Chinese materia medica is sourced from the *Pharmacopoeia of the People’s Republic of China 2020* (Ch.P), TCMSP, ETCM, and TCM Miner. The Ch.P represents the most authoritative codex for traditional Chinese medicine data as an essential component of China’s national drug standards, containing 616 commonly used Chinese herbal medicines. The TCM Miner is an information retrieval platform developed by the Institute of Traditional Chinese Medicine Information of China Academy of Chinese Medical Sciences, collecting Chinese herbal medicine’s basic information, local standards, and national standards.

#### Data of chemical components

Information on the chemical components of Chinese herbal medicine primarily comes from databases such as PubChem, TCMSP, TCM-ID, and HERB. PubChem, developed by the United States National Institutes of Health, is an open-source database that includes information on small molecule compounds’ physicochemical properties, biological activities, toxicities, and patents [14]. A comprehensive search is conducted within the DrugBank database using the obtained component entity information, acquiring additional component entities information, including molecular formulae, SMILES codes, and indications [15].

#### Data of targets

Primary data sources for target entities include databases such as TCMSP, SymMap, SwissTargetPrediction, NCBI Gene, HERB, and others. SwissTargetPrediction predicts molecular targets based on the SMILES code or structural formulas of small molecules [16]. NCBI Gene, a gene data platform developed by the United States National Center for Biotechnology Information, includes extensive gene attribute and entity information [17].

#### Disease data

Disease entity data mainly originates from DisGetNET and GeneCard databases. DisGetNET consolidates human disease-related gene information from various disease databases, including OMIM, HPO, UMLS, and MeSH. GeneCard, a free, comprehensive database available to research institutions, has integrated information from 193 public gene-associated databases as of December 1, 2023, and offers a wealth of transcriptome, genome, and proteome data [18]. In this study, diseases corresponding to pCms were determined based on their leaflets and relevant literature concerning their modern clinical applications. Due to possible inconsistencies in gene or protein IDs from different source databases, manual examination and conversion into official gene IDs and protein Uniprot IDs were performed.

#### Entity relationship and network construction

Integrating entity data from various databases results in three types of entity relationships: pCms–Chinese herbal medicines, Chinese herbal medicine–components, and components–targets. The network module within the vis.js dynamic browser-based database was employed to construct user-friendly networks based on the entity relationships among pCms, Chinese herbal medicines, components, and targets. These networks were then visualized on the database’s corresponding pages.

### Construction of antitumor pCms–component–target network

By using bibliometric approach, preliminary research has identified several antitumor pCms which are widely adopted in GC treatment (Additional file 1). In this study, these antitumor pCms’ names were searched within the databases to obtain their formulation, followed by searching for corresponding chemical component and target data. The target information for GC was acquired by searching the antitumor pCm database using “Gastric Cancer” as the keyword. Utilizing information on pCms, Chinese herbal medicines, components, and targets, the network files and type files were prepared and imported into Cytoscape software for network visualization. This process resulted in the construction of the antitumor pCm–component–target network. Network topology analysis was then conducted, and Degree values were derived for subsequent analyses.

### Construction of PPI network for GC treatment with antitumor pCms

Intersection targets between antitumor pCm targets and GC targets were determined and visualized using a Venn diagram. The intersection targets were analyzed in the STRING database (https://cn.string-db.org/), setting the minimum required interaction score to 0.4, displaying only connected network nodes, and obtaining the.tsv file of the PPI network. This file was then imported into Cytoscape software for PPI network visualization and subsequent network topology analysis, with key targets identified based on Betweenness, Closeness, and Degree values.

### Molecular docking

Initially steps involved obtaining chemical component nodes with a Degree > 100 from the antitumor pCm–component–target network, which were considered as the network’s core components. Key targets were then screened based on the topological parameters of the PPI network. Core components and key targets were assumed to be ligands and receptors, respectively, and used for molecular docking. Molecular docking calculations were performed using Autodock Vina 1.1.2. The binding free energies between core chemical components and key targets were ranked in ascending order. A molecular docking heatmap was generated using the pheatmap package in R, and core chemical components with minimum binding energy levels to bind to the key targets were selected. Docking results were visualized using PyMOL 2.3.2 software.

### Cell lines, cell culture, and experimental drugs

The human GC cell line HGC-27, acquired from Wuhan Procell Life Science & Technology Co., Ltd., was verified by STR identification. Cells were routinely cultured in RPMI-1640 complete medium (Procell, China) at 37 °C, 5% CO_2_, and saturated humidity. Norcantharidin (Purity ≥ 98%) was procured from Shanghai YuanYe Biotechnology Co., Ltd.

### CCK-8 assay

Cells were digested with a 0.25% trypsin solution (containing EDTA) and seeded in 96-well plates at a density of 5.0 × 10^4^ cells/mL, with 100 μL per well and 6 replicates per group for 24 h. For each well, complete medium containing norcantharidin at concentrations of 1.25 μM, 2.5 μM, 5 μM, 10 μM, 20 μM, 40 μM, 80 μM, and 160 μM was added and cultured for an additional 24, 48, and 72 h. At the end of the culture period, 100 μL of CCK-8 diluent (culture medium: CCK-8 reagent = 9:1) was added to each well, incubated in a 37 °C incubator for over 30 min, and the OD value at 450 nm was determined using a microplate reader. A cell viability curve was then plotted.

### Colony formation assay

Cells were digested with a 0.25% trypsin solution (containing EDTA) and seeded in 6-well plates at a density of 5.0 × 10^2^ cells/mL, with 1 mL per well and a 24 h culture period. The control group was provided with complete medium, while treatment groups received complete medium containing norcantharidin at concentrations of 15 μM, 30 μM, and 60 μM. The cells were cultured until macroscopically visible colonies developed in the wells. Once colonies were observed, the cell culture was terminated, and each well was fixed using a 4% PFA solution, stained with 0.1% crystal violet, and counted using ImageJ software.

### Wound healing assay

Cells were treated with a 0.25% trypsin solution (containing EDTA) and seeded in 6-well plates at a density of 5.0 × 10^5^ cells/mL, with 1 mL per well, followed by gentle shaking to ensure even distribution. After 24 h, the control group was provided with serum-reduced medium, and the treatment groups were exposed to serum-reduced medium containing norcantharidin at concentrations of 15 μM, 30 μM, and 60 μM for 48 h. Photos were taken at 0, 12, 24, and 48 h intervals, while wound area calculations were conducted using ImageJ software.

### Transwell migration and invasion assay

For Transwell migration assay, cells were treated with a 0.25% trypsin solution (containing EDTA) and seeded at a density of 3.0 × 10^5^ cells/mL and 100 μL per well in the upper chamber of the Transwell. Serum-free medium (Control group), or serum-free medium containing norcantharidin at concentrations of 15 μM, 30 μM, and 60 μM were added to the upper chamber. Complete medium was added to the lower chamber, and the cells were cultured for 24 h. Finally, cells were fixed with 4% PFA, stained with 0.1% crystal violet staining solution, photographed, and counted using ImageJ software.

For Transwell invasion assay, 60 μL of Matrigel dilution (Matrigel:PBS = 1:8) was added to the upper chamber of the Transwell and placed in the incubator for gel formation. Then, 100 μL of serum-free medium was added to the upper chamber. Cells were digested with a 0.25% trypsin solution (containing EDTA) and seeded at a density of 3.0 × 10^5^ cells/mL with 100 μL per well in the upper chamber. Subsequently, serum-free medium (Control group) or serum-free medium containing norcantharidin at concentrations of 15 μM, 30 μM, and 60 μM were added to the upper chamber. The lower chamber was loaded with complete medium, and cells were cultured for an additional 24 h. Lastly, cells were fixed using 4% PFA, stained with 0.1% crystal violet staining solution, photographed, and counted with ImageJ software.

#### Cell cycle detection

Cells were treated with a 0.25% trypsin solution (containing EDTA) and seeded at a density of 3.0 × 10^5^ cells/mL, with 1 mL per well in 6-well plates, and cultured for 24 h. Following this, the control group received complete medium, while treatment groups were exposed to complete medium with norcantharidin at concentrations of 15 μM, 30 μM, and 60 μM for an additional 48 h. Upon completion, cells were digested with 0.25% trypsin solution (without EDTA), and cell suspensions were collected, centrifuged at 1500 rpm for 5 min, fixed with pre-chilled anhydrous ethanol, and incubated at − 20 °C for 24 h. Cells were resuspended in PBS and processed using the PI staining solution according to kit instructions. Red fluorescence was detected using flow cytometry at an excitation wavelength of 488 nm, and data were analyzed using FlowJo software.

#### Cell apoptosis detection

Cells were treated with a 0.25% trypsin solution (containing EDTA) and seeded at a density of 3.0 × 10^5^ cells/mL, with 1 mL per well in 6-well plates and cultured for 24 h. the control group received complete medium, whereas treatment groups were cultured in complete medium containing norcantharidin at concentrations of 30 μM, 60 μM, and 90 μM for 48 h. After collecting the original culture medium and washing with PBS, cells were digested with 0.25% trypsin solution (without EDTA) and suspensions were collected. Following centrifugation at 600*g* for 5 min, the supernatant was discarded, and cells were processed according to the instructions provided in the Annexin V-FITC/PI Fluorescence Double Staining Cell Apoptosis Detection Kit. Immediate flow cytometry analysis was performed, and data were analyzed using FlowJo software.

#### Transcriptome sequencing and data quality control

PE libraries were prepared following the instructions provided in the mRNA-seq Lib Prep Kit. mRNA was isolated from total RNA using oligo(dT) magnetic beads and fragmented in Abclonal First Strand Synthesis Reaction Buffer. Subsequently, cDNA synthesis was performed using mRNA as a template, and the double-stranded cDNA fragments were ligated to adapter sequences. Library fragments were amplified using PCR, with library quality assessed via Agilent Bioanalyzer 4150. Alkaline denaturation transformed them into single-stranded libraries, followed by paired-end sequencing on the NovaSeq 6000 platform.

FastQC 0.11.9 software assessed raw data quality, with Trim_galore 0.6.6 software removing adapter sequences and filtering low-quality data (bases with quality scores ≤ 25 accounting for over 60% of reads) and reads with an N ratio > 5%. This procedure yielded Clean Reads for subsequent analysis. Clean Reads were aligned to reference genome data using HISAT2 software, generating Mapped Reads for further investigation. The FeatureCounts tool calculated the read counts for each gene, with FPKM values determined based on gene length.

#### DEGs expression analysis and enrichment analysis

Untreated HGC-27 cells served as the control group, while HGC-27 cells treated with norcantharidin constituted the treatment group. The DESeq2 package in R software was employed for inter-group DEGs expression analysis, with screening criteria set as |log2FoldChange|> 1 and *p*-value > 0.05 to identify DEGs before and after norcantharidin intervention on GC.

DEGs, downregulated DEGs, and upregulated DEGs were analyzed separately for biological processes (BP), cellular components (CC), molecular functions (MF), and KEGG pathways using the David database (https://david.ncifcrf.gov/), GO database (http://geneontology.org/), and KEGG database (http://www.kegg.jp). The hypergeometric distribution algorithm calculated the significance of DEGs within corresponding GO terms and KEGG pathways. Entries with *p*-value < 0.05 were selected for analysis of their biological significance and were used for subsequent key gene identification.

#### Key target acquisition

Jvenn (http://www.bioinformatics.com.cn/static/others/jvenn/) online plotting tool was utilized to obtain the intersection of norcantharidin upregulated, downregulated GC DEGs, and the DEGs in GC patients from the TCGA-STAD dataset [19]. These intersections were subsequently defined as key molecules in norcantharidin treatment of GC.

Gene set enrichment analysis was performed on the key molecules from norcantharidin intervention in GC using the Hallmark gene set from the MsigDB database (https://www.gsea-msigdb.org/gsea/msigdb) [20–22]. The hypergeometric distribution algorithm calculated the enrichment significance of each DEGs within the Hallmark gene set, with entries featuring a *p*-value < 0.05 used for selecting the core molecules.

Using the STRING database, the PPI network of key molecules involved in norcantharidin intervention in GC was constructed, setting the required minimum interaction score = 0.4. This network was imported into Cytoscape software for PPI network visualization, with the Cytohubba plugin revealing the key modules and core molecules within the network [23, 24]. Appropriate gene sets were subsequently selected based on enrichment analysis results; intersections were used to screen for key targets.

## Results

### Antitumor pCm database

**Fig. 2:**
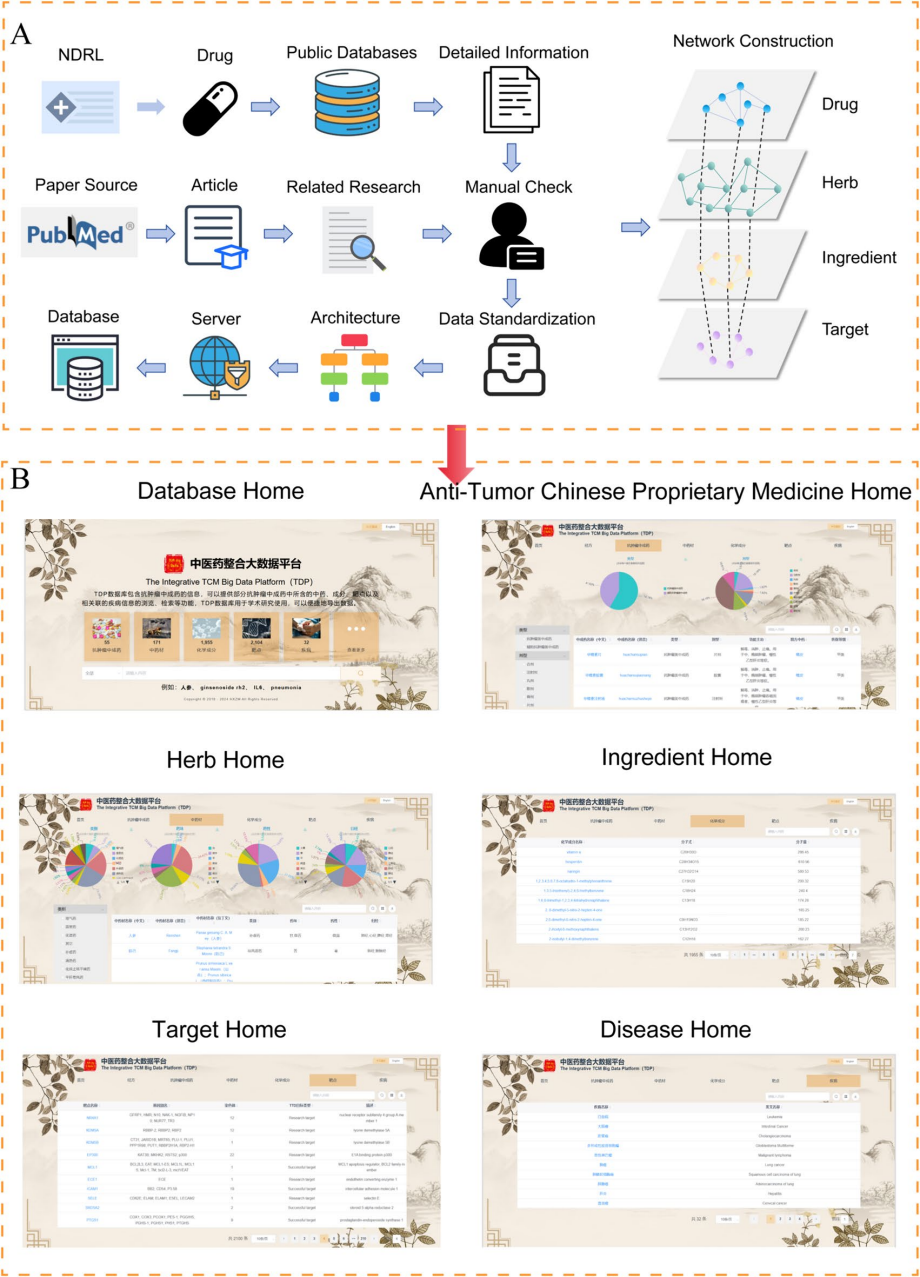
Schematic diagram of the database. **A** Schematic diagram of the database construction process. **B** Schematic diagram of the database interface

**Fig. 3:**
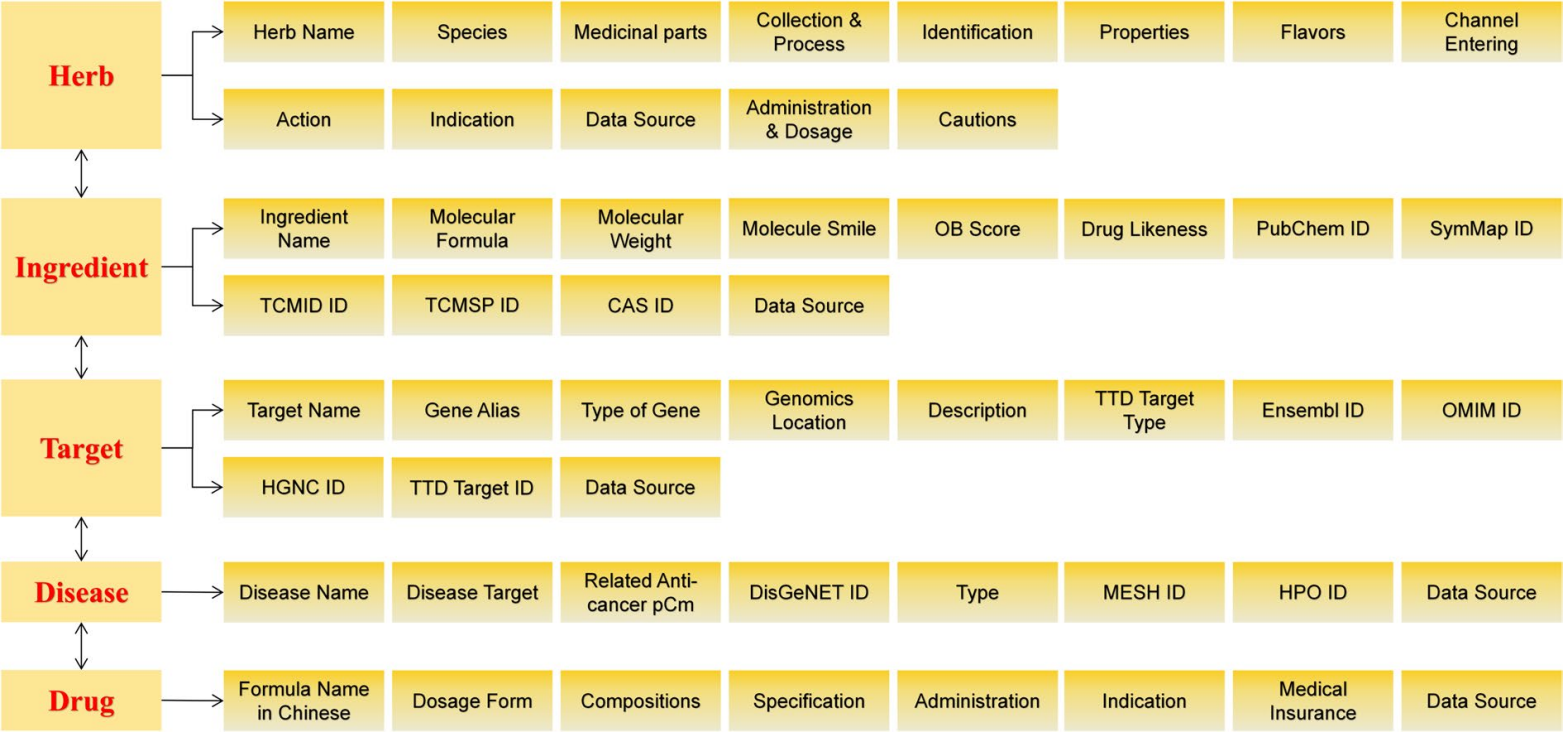
Field names in the database

In this study, we retrieved 55 antitumor pCms from NRDL; we collected data on 171 Chinese herbal medicines and their related information from sources such as the Ch.P, TCMSP, ETCM, and TCM Miner. The database is available at http://zyy.intelligence-media.com/index. We organized information on 1955 chemical components from PubChem, SymMap, TCMSP, TCM-ID, and HERB; collected 2104 targets from TCMSP, SymMap, SwissTargetPrediction, NCBI Gene, and HERB; and obtained data on 32 drug-related diseases from DisGetNET and GeneCard databases (Figs. 2, 3, Table 1).

**Table 1:** Sources of information in the database

Entity	Number	Origin
pCm	55	NRDL
Chinese herbal medicine	171	Ch.P, TCMSP, ETCM, TCM-Miner
Chemical compound	1955	PubChem, SymMap, TCMSP, TCM-ID, HERB
Target	2104	TCMSP, SymMap, SwissTargetPrediction, NCBI Gene, HERB
Disease	32	DisGetNET, GeneCard

pCm: proprietary Chinese medicine; NRDL: China’s *Catalogue of Drugs for Basic National Medical Insurance/Employment Injury Insurance/Birth Insurance (2022)*; Ch.P: *Pharmacopoeia of People’s Republic of China 2020*

**Fig. 4:**
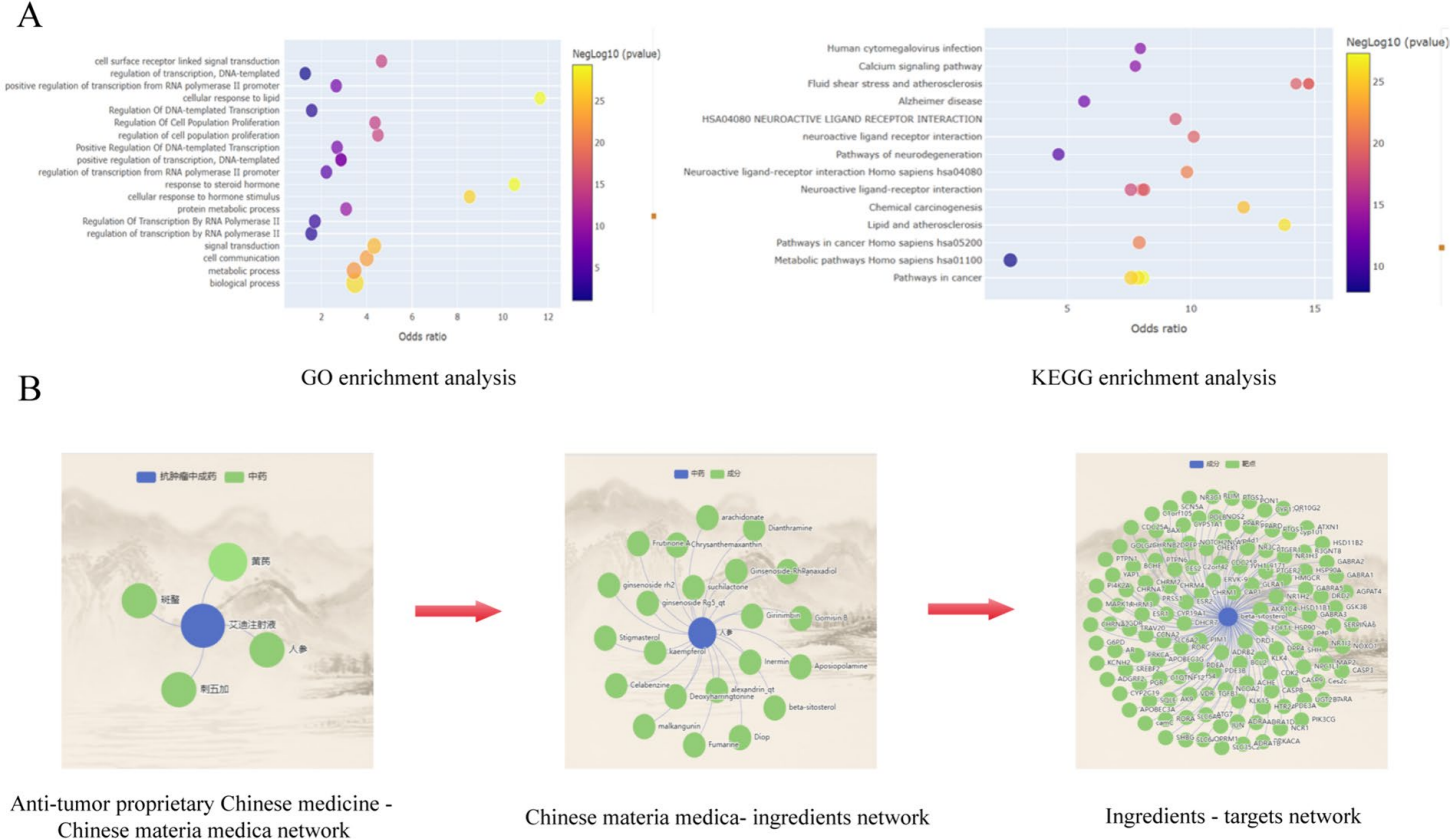
Schematic diagram of the database functions. **A** Enrichment analysis. **B** Network diagram

This study utilizes a B/S architecture, develops a relational MySQL database based on CHINER meta-number modeling, employs Navicat Premium for daily database management and maintenance, and adopted Vue and Spring frameworks for front-end and back-end development. The Apache MyBatis is used to create the database persistence layer, which maps raw data into the database. We utilized the Python language to perform a GO function enrichment analysis and KEGG pathway enrichment analysis on pCms and targets (Fig. 4A). We constructed an antitumor pCm–Chinese herbal medicine network, Chinese herbal medicine–component network, and component–target network based on the entity associations between pCms, Chinese herbal medicines, components, and targets (Fig. 4B).

Users may search by entity information on the database homepage and various module pages, browse detailed and related data through different interactive links, and conduct GO or KEGG enrichment analysis for pCms and targets. A secondary network display is provided on the detailed information pages of pCms, Chinese herbal medicines, components, and targets, allowing users to download data according to their requirements.

### Antitumor pCm–component–target network

**Fig. 5:**
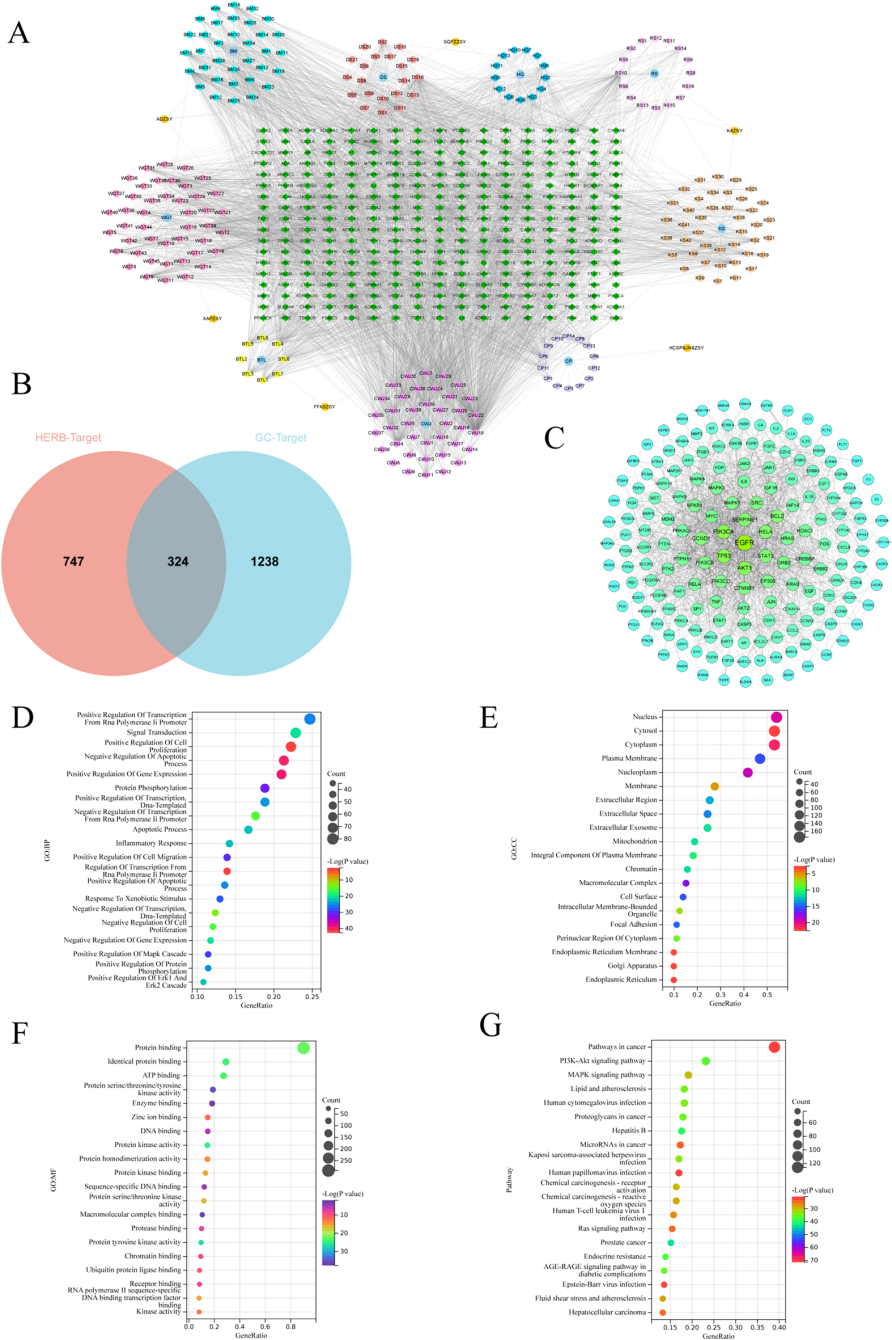
Network pharmacological analysis of antitumor proprietary Chinese medicine. **A** Antitumor proprietary Chinese medicine–component–target network diagram. Hexagonal nodes represent antitumor proprietary Chinese medicine, octagonal nodes represent constituent Chinese medicines in the prescription, circular nodes represent chemical components, diamond nodes represent drug targets. **B** Intersection target Venn diagram. **C** PPI network diagram of potential targets for antitumor proprietary Chinese medicine treating gastric cancer, node size and color correspond to degree values, with larger nodes and deeper colors indicating higher degree values. **D–G** Enrichment analysis charts. **D** GO functional enrichment analysis: biological process (BP); **E** GO functional enrichment analysis: cellular component (CC); **F** GO functional enrichment analysis: molecular function (MF); **G** KEGG pathway enrichment analysis

The antitumor pCms mentioned above comprise 249 chemical components and 1071 targets. The antitumor pCm–component–target network generated by Cytoscape software consists of 533 nodes and 3766 edges. The connections between nodes represent the relationships between chemical components and targets (Fig. 5A). Based on this network, we identified chemical component nodes with Degree values > 100: astragaloside A, ginsenoside Rg3, cis-9-cis-12-octadecadienoic acid, bufothionine, oxymatrine, lauric acid, norcantharidin, isofraxidin, and luteolin (Table 2).

**Table 2:** Information on chemical components with Degree values > 100

Component	CAS ID	Degree
Luteolin	491-70-3	114
Astragaloside A	83,207-58-3	132
Norcantharidin	5442-12-6	105
Ginsenoside Rg3	11,019-45-7	121
Isofraxidin	486-21-5	111
Cis-9-cis-12-octadecadienoic acid	287,111-25-5	101
Bufothionine	16,369-08-7	102
Oxymatrine	16,837-52-8	116
Lauric acid	143-07-7	103

### PPI network of antitumor pCms for GC treatment

We extracted 1562 GC targets from the anti-tumor pCm database. By employing R language, we intersected these targets with the 1071 targets of 9 Chinese herbal medicines, resulting in 324 intersecting targets (Fig. 5B). We then imported the analysis results from the STRING database into Cytoscape to generate the PPI network graph, which comprises 175 nodes and 1,336 edges (Fig. 5C). Through the screening based on betweenness, closeness, and degree values in the PPI network, we identified key targets: EGFR, PIK3CA, TP53, AKT1, SERPINE1, and BCL2 (Table 3).

**Table 3:** Network topology information of key targets

Uniprot ID	Targets	Name	Betweenness	Closeness	Degree
P00533	EGFR	Epidermal growth factor receptor	0.17772604	0.47796610	77
P42336	PIK3CA	Phosphatidylinositol-4,5-Bisphosphate 3-Kinase Catalytic Subunit Alpha	0.06497753	0.44339623	54
P04637	TP53	Cellular tumor antigen p53	0.07070254	0.46381579	52
P31749	AKT1	AKT Serine/Threonine Kinase 1	0.06479168	0.46457990	50
P05121	SERPINE1	Serine protease inhibitor clade E member 1	0.05551783	0.44131455	43
P10415	BCL2	B cell lymphoma 2	0.04999378	0.45337621	41

### Enrichment analysis of antitumor pCm targets for GC treatment

We performed GO function enrichment analysis and KEGG enrichment analysis on the 175 nodes within the PPI network. The GO function enrichment analysis results revealed that relevant targets were primarily enriched in BPs such as signal transduction, apoptotic processes, and inflammatory responses (Fig. 5D); CCs like nuclei, cytoplasm, and plasma membranes (Fig. 5E); MFs including ATP binding, protein kinase activity, and DNA binding (Fig. 5F); as well as signaling pathways like cancer pathways, PI3K-AKT signaling pathways, and MAPK signaling pathways (Fig. 5G).

### Molecular docking

**Fig. 6:**
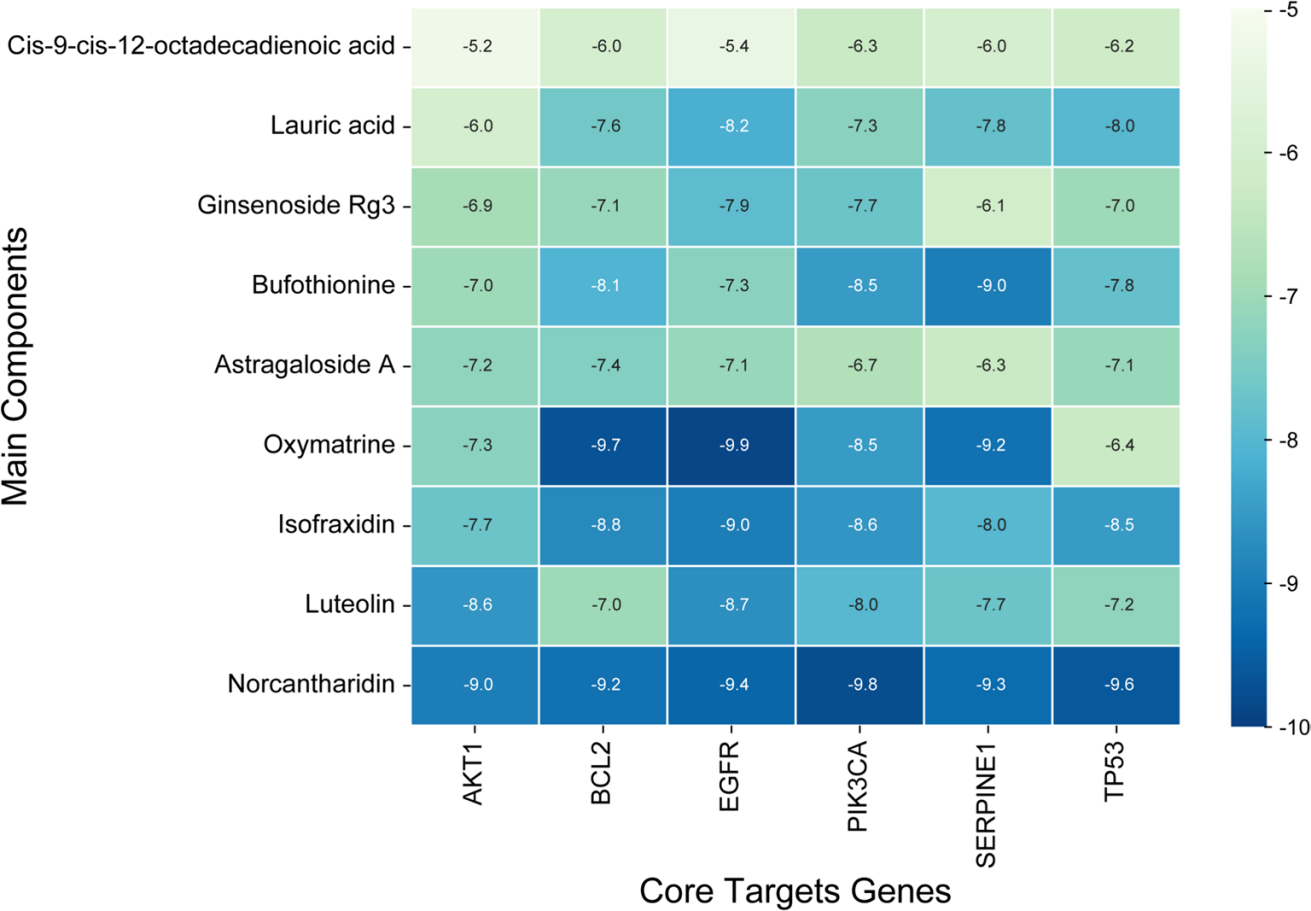
Heat map of molecular docking

**Fig. 7:**
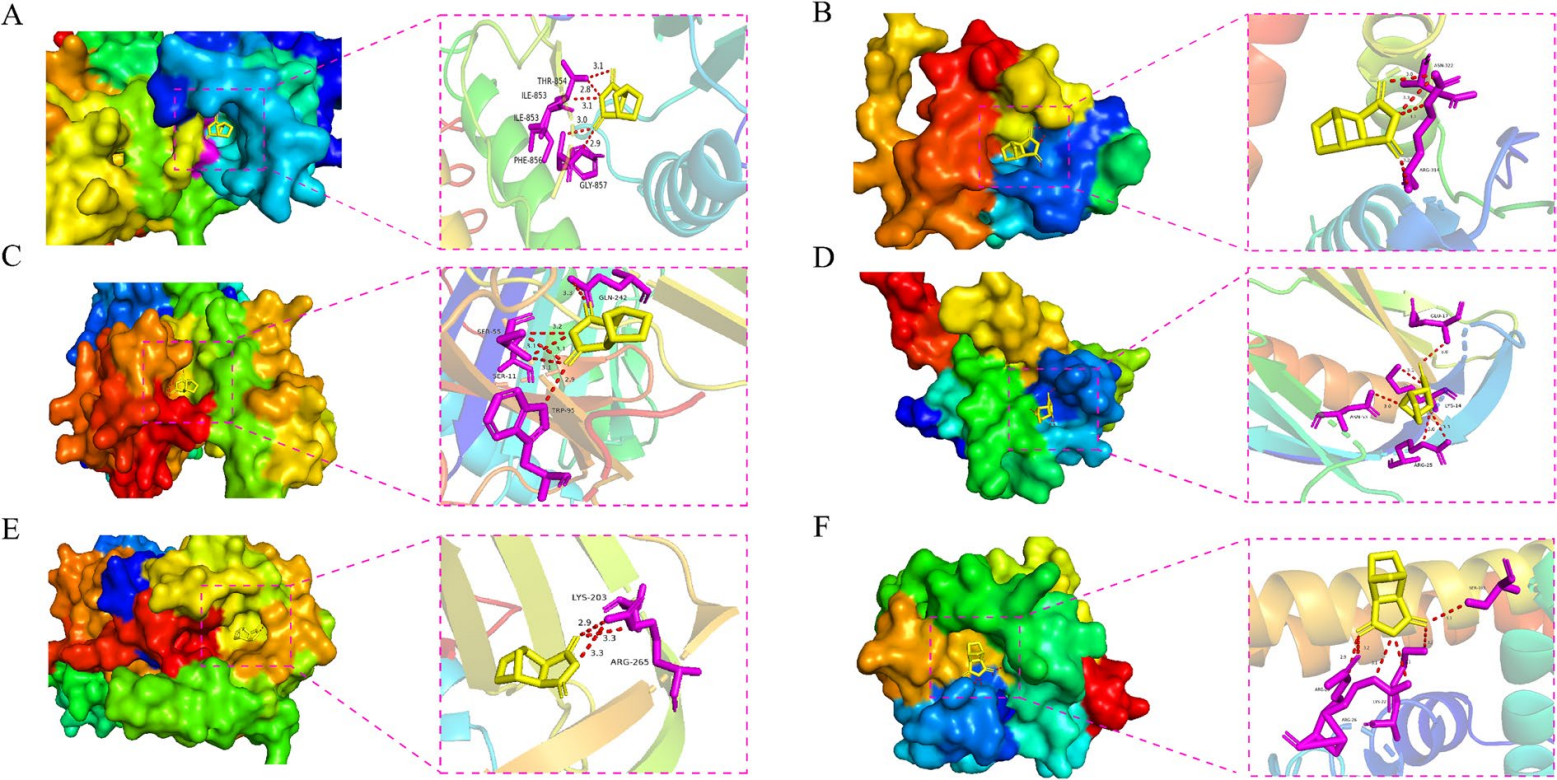
Molecular docking diagrams. **A** NCTD acting on EGFR (binding energy: − 9.4 kcal/mol). **B** NCTD acting on TP53 (binding energy: − 9.6 kcal/mol). **C** NCTD acting on PIK3CA (binding energy: − 9.8 kcal/mol). **D** NCTD acting on AKT1 (binding energy: − 9.0 kcal/mol). **E** NCTD acting on SERPINE (binding energy: − 9.3 kcal/mol). **F** NCTD acting on BCL2 (binding energy: − 9.2 kcal/mol). NCTD: norcantharidin

We conducted molecular docking with the chemical components that were screened from the antitumor pCm–component–target network and crucial targets obtained from the PPI network analysis. All molecular docking results were visualized using heat maps (Fig. 6, Table 4). We eventually discovered that norcantharidin exhibited the lowest binding energy with six core targets among all crucial chemical components, and visualized its molecular docking results (Fig. 7).

**Table 4:** Molecular docking results of chemical components and key targets

Chemical component	Binding energy(kcal/mol)					
	AKT1 PDB ID	BCL2 PDB ID	EGFR PDB ID	PIK3CA PDB ID	SERPINE1 PDB ID	TP53 PDB ID
	(7MYX)	(6G18)	(8A27)	(7L1C)	(4KDS)	(8SWJ)
Luteolin	− 8.6	− 7.0	− 8.7	− 8.0	− 7.7	− 7.2
Astragaloside A	− 7.2	− 7.4	− 7.1	− 6.7	− 6.3	− 8.0
Norcantharidin	− 9.0	− 9.2	− 9.4	− 9.8	− 9.3	− 9.6
Ginsenoside Rg3	− 6.9	− 7.1	− 7.9	− 7.7	− 6.1	− 7.0
Isofraxidin	7.7	− 8.8	− 9.0	− 8.6	− 8.0	− 8.5
Cis-9-cis-12-octadecadienoic acid	− 5.2	− 6.0	− 5.4	− 6.3	− 6.0	− 6.2
Bufothionine	− 7.0	− 8.1	− 9.3	− 8.5	− 9	− 7.8
Oxymatrine	− 7.3	− 9.7	− 9.9	− 9.7	− 9.2	− 8.0
Lauric acid	− 6.0	− 7.6	− 8.2	− 7.3	− 7.8	− 8.0

### Norcantharidin inhibits GC cell proliferation

**Fig. 8:**
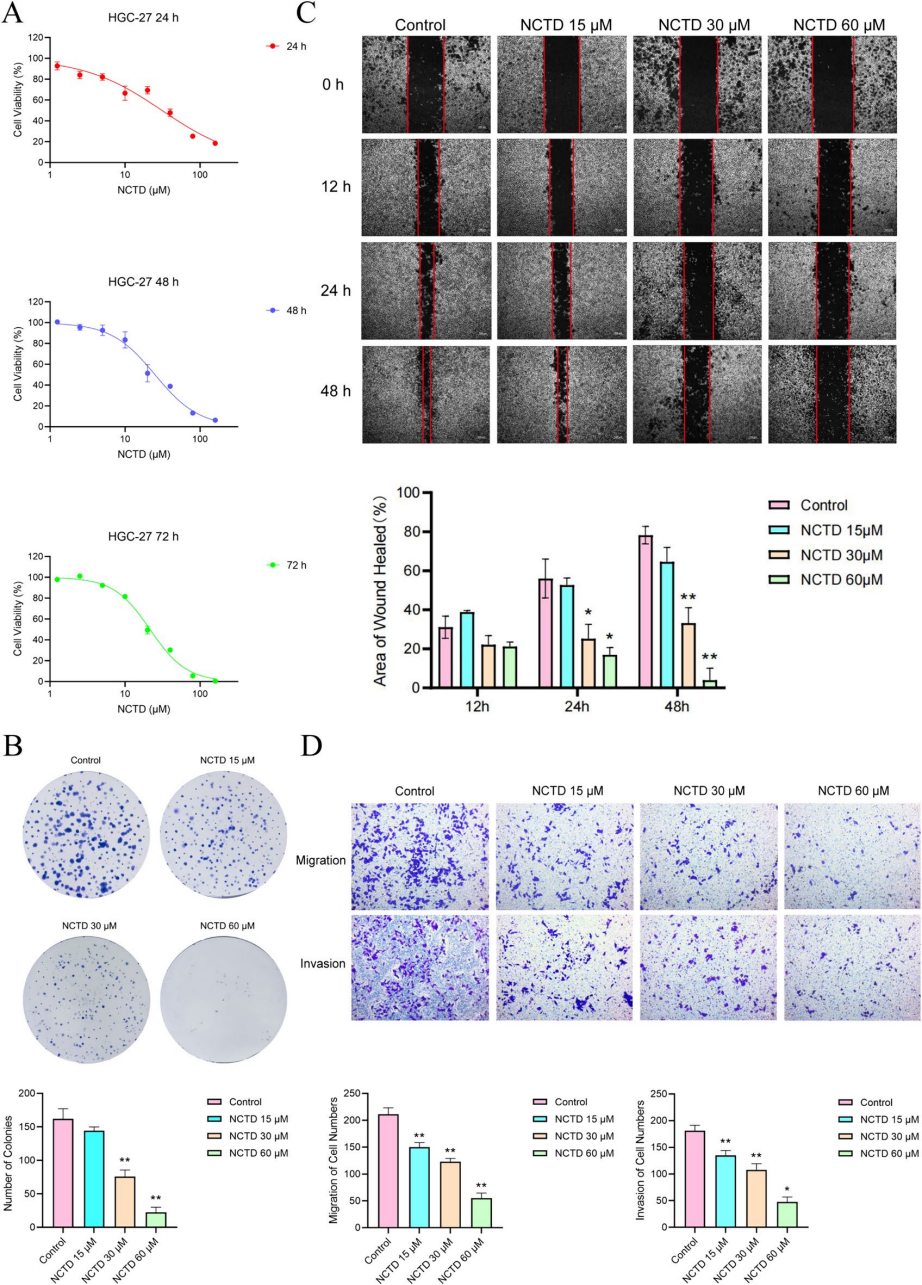
NCTD inhibits HGC-27 cell proliferation, migration, and invasion. **A** CCK8 assay for NCTD inhibition of gastric cancer cell proliferation, determining subsequent experimental drug concentrations. **B** Colony formation assay for NCTD inhibition of gastric cancer cell proliferation. **C** Wound healing assay for NCTD intervention in gastric cancer cell migration. **D** Transwell assay for NCTD intervention in gastric cancer cell migration and invasion. Data are represented as n = 3, $\overline{x}$± SD. Compared to the control group (Control), *p < 0.05, **p < 0.01. NCTD: Norcantharidin

CCK8 results demonstrated that norcantharidin suppressed the proliferation of human GC HGC-27 cells in a dose- and time-dependent manner, with 24 h IC_50_ values = 30.26 ± 0.78 μmol/L, 48 h IC_50_ values = 26.89 ± 1.49 μmol/L, and 72 h IC_50_ values = 23.33 ± 1.27 μmol/L. In this study, we used the 24 h IC_50_ value as the medium dose and conducted subsequent experiments with three concentrations: 15, 30, and 60 μmol/L (Fig. 8A).

### Norcantharidin inhibits GC cell migration and invasion

A strong connection exists between cell scratch healing rates and migration ability. Wound healing experiments results reveal that norcantharidin, at concentrations of 30 μmol/L and 60 μmol/L, significantly inhibits the migration ability of human GC HGC-27 cells in a concentration-dependent manner (Fig. 8C). Cells stained with crystal violet at the bottom of the Transwell chamber that have passed through the polycarbonate membrane are considered to have migration ability. Transwell migration results demonstrate that norcantharidin significantly suppresses the migration ability of human GC HGC-27 cells in a concentration-dependent manner (Fig. 8D).

Cells at the Transwell chamber’s bottom stained with crystal violet could dissolve the Matrigel basement membrane and pass through the polycarbonate membrane are considered to possess invasive capabilities. Transwell invasion results reveal that norcantharidin considerably hinders the invasion ability of human GC HGC-27 cells in a concentration-dependent manner (Fig. 8D).

### Norcantharidin induces G2/M phase arrest in GC cells

**Fig. 9:**
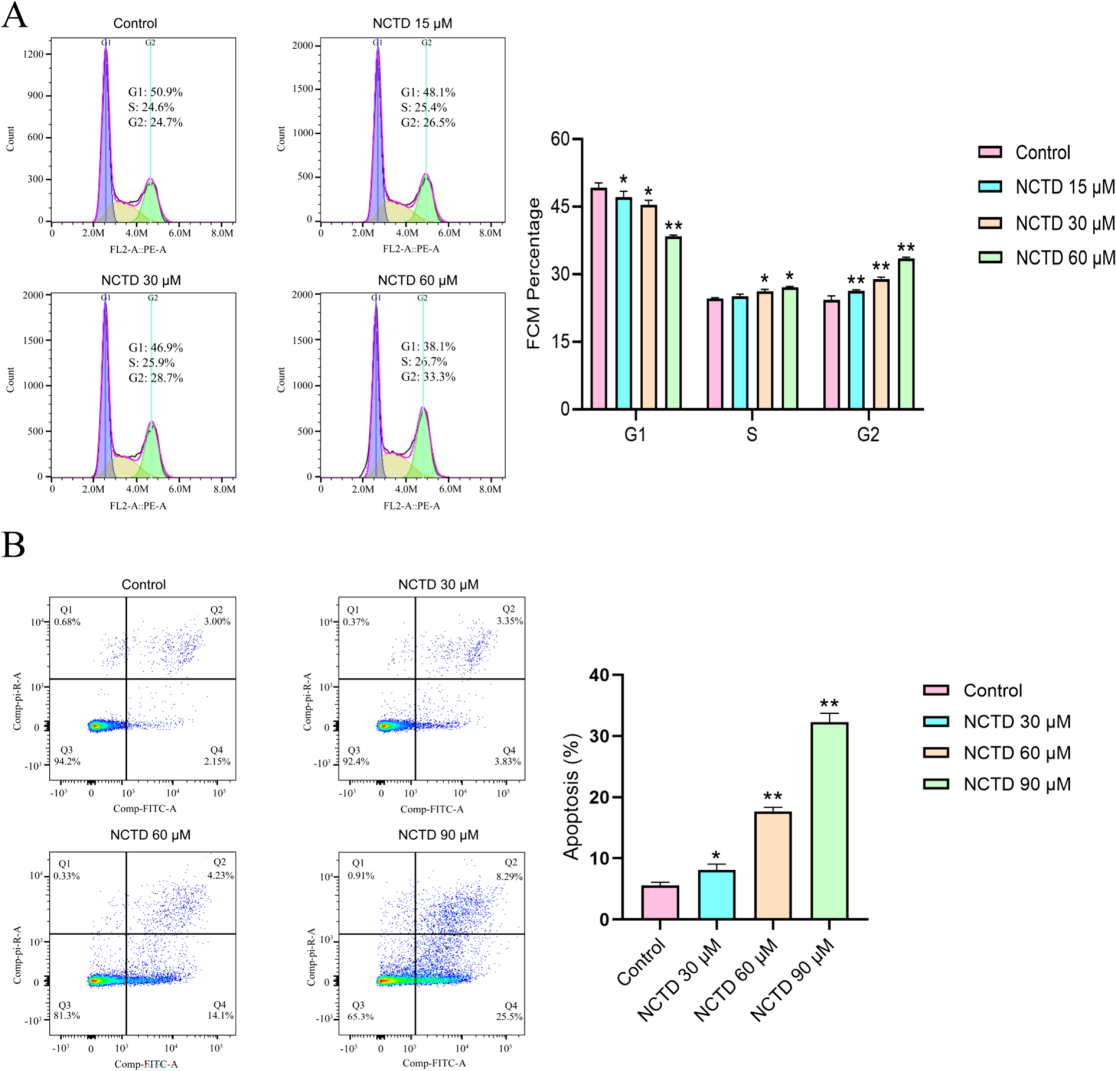
NCTD Induces HGC-27 Cell Cycle Arrest and Apoptosis. **A** Flow cytometry detection of PI-stained cell cycle distribution. **B** Flow cytometry detection of PI and Annexin V double-stained cell apoptosis. Data are represented as n = 3, $\overline{x}$± SD. Compared to the control group (Control), *p < 0.05, **p < 0.01. NCTD: Norcantharidin

Compared to the control group, the norcantharidin treatment group experienced a significant reduction in the number of G1 phase cells, a considerable increase in G2 phase cells, and an increase in S phase cells, demonstrating a dose-dependent relationship. These findings suggest that norcantharidin impacts GC cell DNA synthesis, leading to G2/M phase arrest (Fig. 9A).

### Norcantharidin induces GC cell apoptosis

Compared to the control group, the norcantharidin treatment group showed a significant increase in the number of apoptotic cells. The number of both early apoptotic cells (Annexin V-FITC positive, PI negative) in the Q4 quadrant and late apoptotic cells (Annexin V-FITC positive, PI positive) in the Q2 quadrant rose significantly, exhibiting a dose-dependent relationship (Fig. 9B).

#### Norcantharidin regulates proliferation and migration-related genes in GC cells

**Fig. 10:**
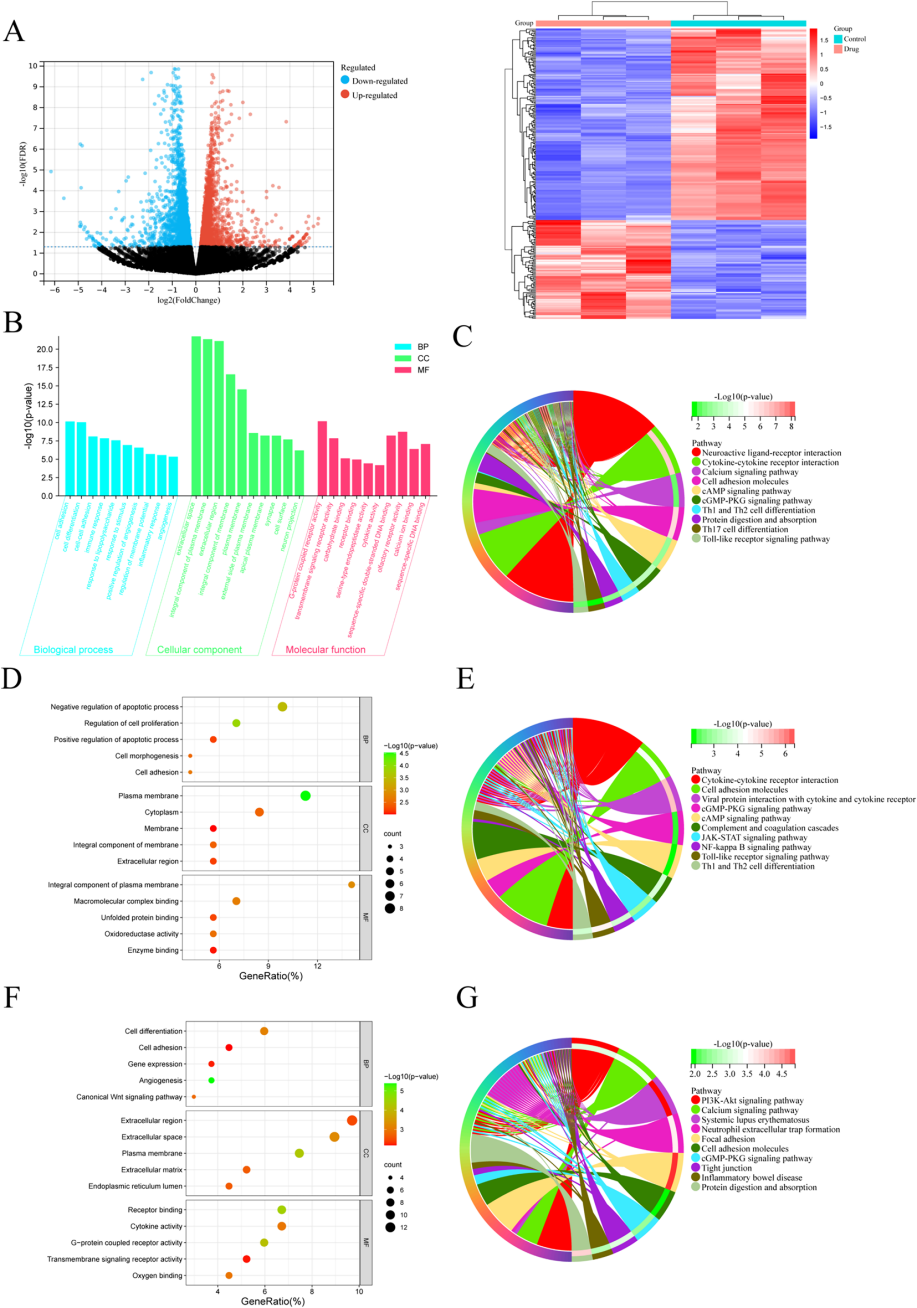
NCTD modulates HGC-27 cell proliferation and migration-related genes. **A** Heatmap and volcano plot of DEGs in NCTD-treated HGC-27 cells. **B** GO enrichment analysis of DEGs in NCTD-treated HGC-27 cells. **C** KEGG enrichment analysis of DEGs in NCTD-treated HGC-27 cells. **D** GO enrichment analysis of upregulated DEGs in NCTD-treated HGC-27 cells. **E** KEGG enrichment analysis of upregulated DEGs in NCTD-treated HGC-27 cells. **F** GO enrichment analysis of downregulated DEGs in NCTD-treated HGC-27 cells. **G** KEGG enrichment analysis of downregulated DEGs in NCTD-treated HGC-27 cells. NCTD: Norcantharidin

We employed the ggplot2 package in R software to construct a volcano plot of DEGs, and the pheatmap package to produce a heatmap of DEGs (Fig. 10A). Analyses revealed that norcantharidin treatment influenced 205 DEGs in GC, with 71 upregulated genes and 134 downregulated genes.

The enrichment analysis of DEGs in GC cells treated with norcantharidin primarily focuses on BPs such as cell adhesion, cell differentiation, inflammatory response, response to stimulus, and positive regulation of angiogenesis; CCs such as extracellular space, plasma membrane, and cell surface; MFs include G-protein-coupled receptor activity, transmembrane signaling receptor activity, receptor binding, and serine-type endopeptidase activity; and signaling pathways such as neuroactive ligand-receptor interaction, calcium signaling pathway, and cell adhesion molecules (Fig. 10B, C).

Enrichment analysis of upregulated DEGs mainly targets BPs like negative regulation of apoptotic process, regulation of cell proliferation, and cell adhesion; CCs like plasma membrane, cytoplasm, and extracellular region; MFs covering macromolecular complex binding, unfolded protein binding, and oxidoreductase activity; and signaling pathways such as cell adhesion molecules, JAK-STAT signaling pathway, and NF-κB signaling pathway (Fig. 10D, E).

Enrichment analysis of downregulated DEGs predominantly involves BPs like cell differentiation, cell adhesion, and angiogenesis; CCs incorporating the extracellular matrix and endoplasmic reticulum lumen; MFs such as transmembrane signaling receptor activity, cytokine activity, and oxygen binding; and signaling pathways including PI3K-AKT signaling pathway, calcium signaling pathway, and focal adhesion (Fig. 10F G).

#### Norcantharidin regulates SERPINE1, a key molecule impacting GC metastasis

**Fig. 11:**
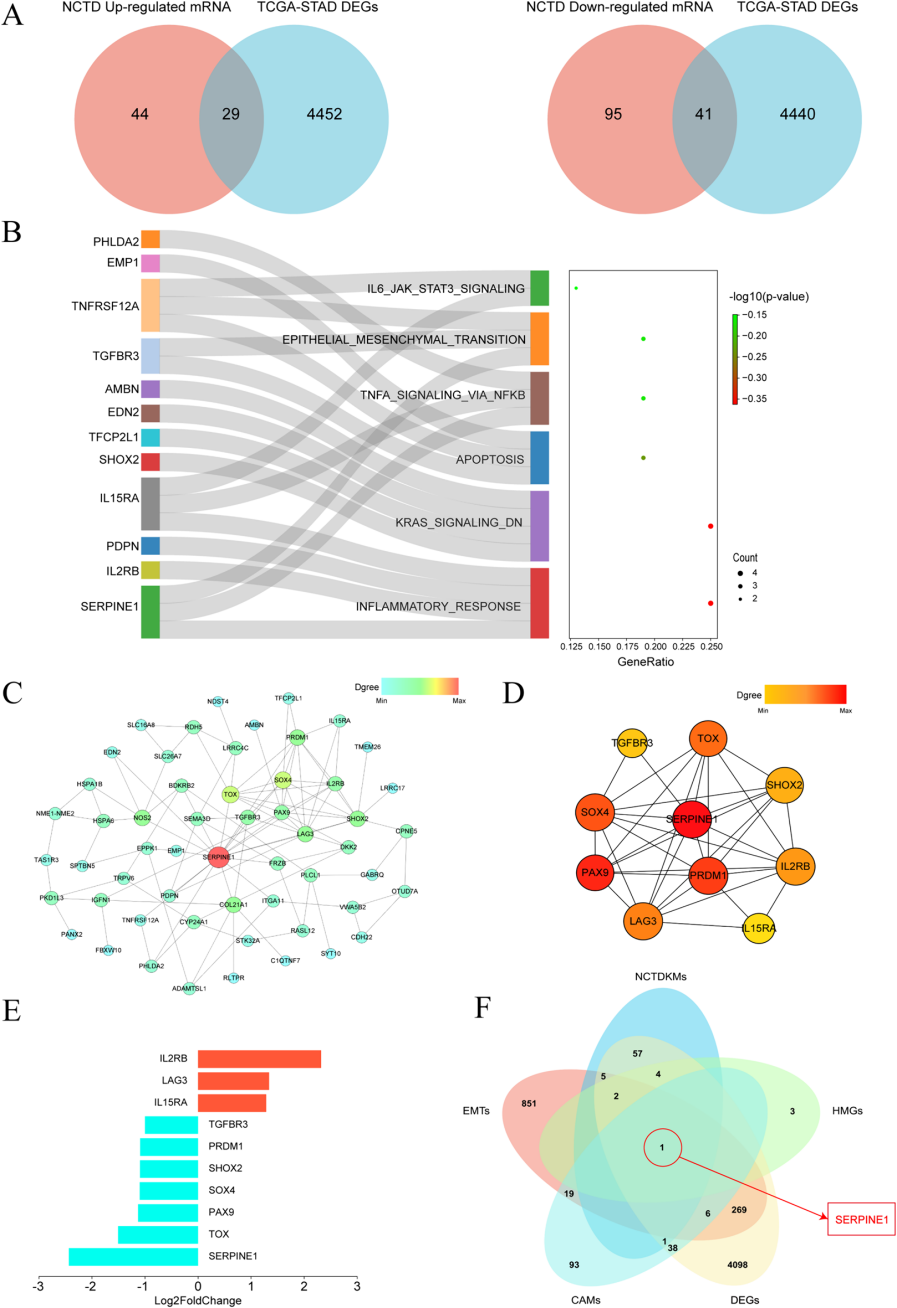
NCTD regulates the key target SERPINE1 affecting gastric cancer metastasis. **A** Venn diagram of key molecules in NCTD-regulated HGC-27 cells. **B** Gene set enrichment analysis of key molecules. **C** PPI network diagram of key molecules. **D** CytoHubba module analysis to identify core molecules. **E** Differential expression levels of core molecules’ mRNA in the CytoHubba module network for NCTD-treated gastric cancer. **F** Venn diagram for screening key targets in NCTD-treated gastric cancer. NCTD: Norcantharidin

In this study, we identified 70 crucial intersection molecules influenced by norcantharidin treatment for GC, including 29 upregulated and 41 downregulated molecules. We visualized these results using Venn diagrams (Fig. 11A). Norcantharidin’s intervention in GC primarily affects gene sets related to epithelial-mesenchymal transition (EMT), inflammatory response, and cell apoptosis, which are associated with malignant biological behaviors such as GC cell proliferation and metastasis. We employed bubble charts combined with Sankey diagrams to visualize gene set enrichment results (Fig. 11B).

The PPI network of key molecules comprises 55 nodes and 107 edges, with SERPINE1 being the node with the largest Degree value (Fig. 11C). The key module network obtained through the CytoHubba plugin contains 10 nodes and 33 edges, with SERPINE1 having the greatest number of connections (Fig. 11D). The 10 core molecules of the key module network, as identified by CytoHubba, include SERPINE1, TGFR3, TOX, SHOX2, SOX4, PAX9, PRDM1, IL2RB, LAG3, and IL15RA. We visualized their differential expression levels (Fig. 11E).

**Fig. 12:**
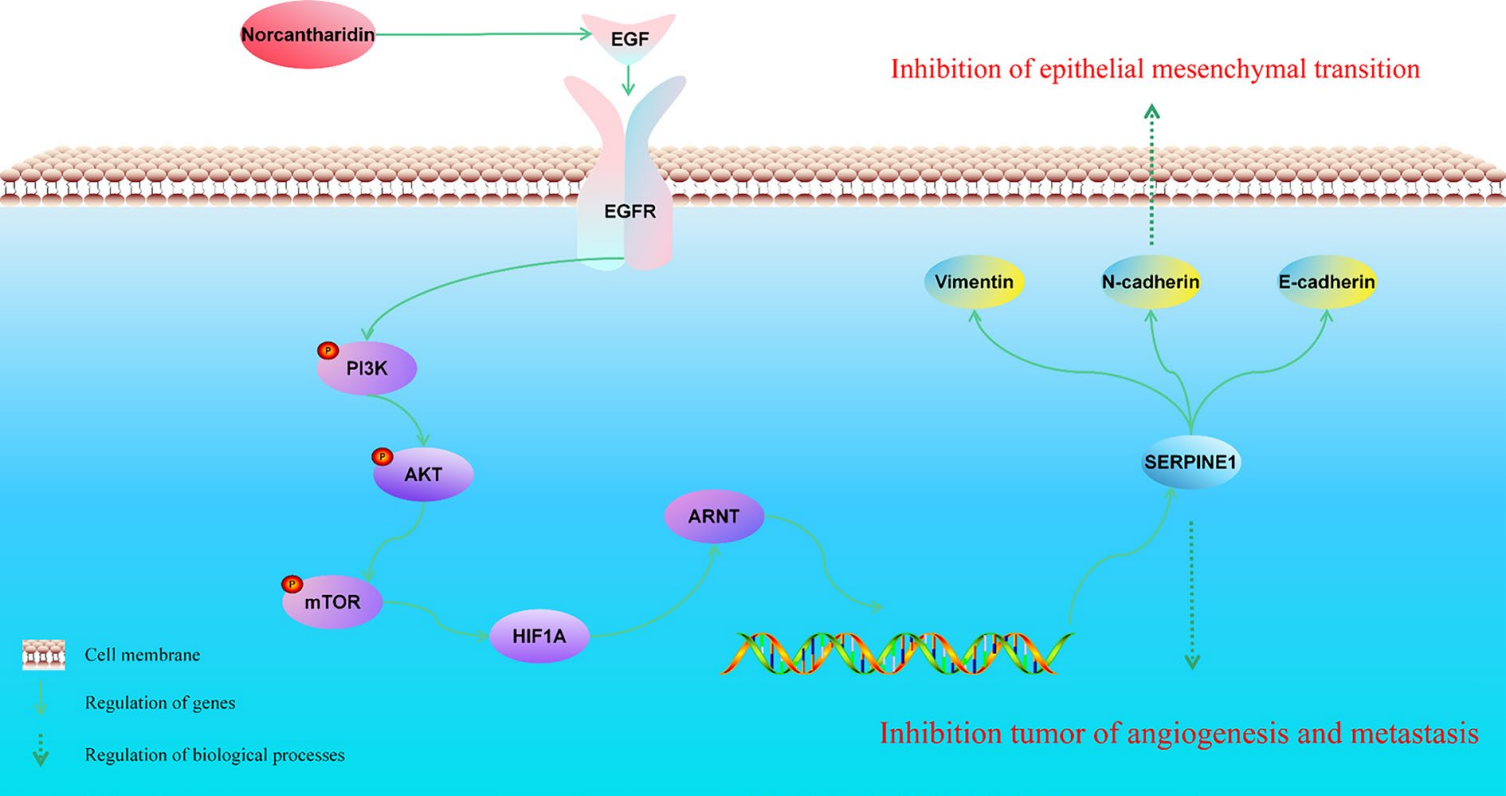
Mechanism of action for norcantharidin treating gastric cancer

These findings suggest that norcantharidin can interfere with malignant biological behaviors such as proliferation and metastasis of GC. Additionally, GO and KEGG enrichment analysis results reveal that norcantharidin mainly affects BPs like cell adhesion, EMTs, and cell apoptosis. Consequently, we selected genes related to the cell adhesion molecule (CAMs) entries in the KEGG database, DEGs in patients’ normal tissues and tumor tissues from the TCGA-STAD dataset (TCGA-DEGs), EMT gene sets (EMTs) from the MSigDB Hallmark gene set (Hallmark-EMTs), targets intersecting between norcantharidin and GC (NCTDKMs), and core molecules of the key module network (HMGs) to identify intersections. Ultimately, we identified the key target SERPINE1 for GC intervention by norcantharidin (Fig. 11F). The mechanism of action for norcantharidin treating GC is depicted in Fig. 12.

We investigated the biological functions of SERPINE1 using bioinformatics analysis. The results indicated that SERPINE1 is highly expressed in GC and various other tumor tissues (Additional file 2). Moreover, SERPINE1 expression negatively correlates with patient overall survival, while positively correlates with tissue pathological staging (Additional file 3). Single and multiple Cox regression analyses revealed that SERPINE1 could serve as a prognostic biomarker for GC (Additional file 4). These findings suggest that norcantharidin may affect GC metastasis by influencing the EMT process, while its critical target, SERPINE1, holds potential as a biomarker for the diagnosis and prognosis of GC.

## Discussion

This study integrates multiple databases related to Chinese herbal medicine, chemical components, targets, and diseases, drawing on antitumor pCms from NRDL. Entity data is consolidated and filtered following the “entity and relationship” and “knowledge interaction” models. Using CHINER meta-element modeling, we established an antitumor pCm database, enabling the query, storage, and visualization of entity information through the MySQL database. This database allows users to search for information on Chinese herbal medicine, components, targets, and diseases by name while navigating detailed and related data through various interactive links. Additionally, the database offers enrichment analysis tools and entity network visualization to a certain extent, catering to users’ analytical needs. The construction of this antitumor pCm database not only lays the groundwork for the subsequent research presented in this paper but also addresses a significant gap in existing TCM databases. This development facilitates more in-depth research on anti-tumor pCms included in the medical insurance catalog.

Network pharmacology enables the effective analysis and processing vast amounts of biological and medical data, interpreting relationships between drugs and diseases from a systems-level and biological network perspective. Molecular docking technology simulates interactions between small-molecule drug ligands and large-molecule protein receptors, significantly advancing new drug development [25]. Chinese medicines, with their multi-component and multi-target properties, integrate seamlessly with network pharmacology and molecular docking technology. Network pharmacology and molecular docking results indicate that norcantharidin is one of the effective components in antitumor pCm for treating GC. This study further examines the efficacy of norcantharidin for GC intervention at the cellular level. Cell experiments reveal that norcantharidin can inhibit GC cell proliferation, migration, and invasion while affecting the synthesis of the GC cell cycle and promoting apoptosis. These findings also support the reliability of network pharmacology and molecular docking prediction results.

To clarify the mechanism of norcantharidin intervention in GC, we employed transcriptome sequencing technology in this study to analyze changes in DEGs before and after the treatment in GC cells. Abnormally expressed molecules were identified post-drug intervention, and enrichment analysis was conducted. This analysis demonstrated that these DEGs were primarily enriched in BPs such as cell adhesion, cell migration, and inflammatory response. Crucial pathways include the PI3K-AKT signaling pathway, NF-κB signaling pathway, JAK-STAT signaling pathway, TNF-α signaling pathway, and EMT-related signaling pathways, which are mainly associated with GC metastasis and proliferation. This finding suggests that norcantharidin may inhibit GC metastasis by intervening in the EMT process. We construct a PPI network using the intersection of DEGs between norcantharidin-treated GC cells and the TCGA-STAD patient dataset. Network topology analysis identified core molecules of norcantharidin intervention in GC patients as SERPINE1, SHOX2, SOX4, and PRDM1.

Serine protease inhibitor clade E member 1 (SERPINE1) is the primary inhibitor of tissue plasminogen activator and urokinase. It plays a role in pathological processes, such as tumor metastasis and tissue fibrosis, and exhibits a wide range of physiological activities. Studies have demonstrated its association with the poor prognosis of various cancers, including GC, colorectal cancer, non-small cell lung cancer, and breast cancer [26–28]. SERPINE1 affects tumor progression by influencing tumor proliferation, migration, and apoptosis. For example, SERPINE1 upregulates cell cycle protein CDK4, accelerates tumor cell growth cycle, resists fibrinolysis, indirectly promotes tumor cell growth, fosters proliferation, inhibits adhesion between tumor cells and vitronectin, and stimulates tumor cell migration to other extracellular matrices [29, 30]. Suppressing SERPINE1 expression inhibits GC cell migration and invasion, an effect that may result from SERPINE1 regulating the expression of VEGF and IL-6 through the VEGF signaling pathway and the JAK-STAT3 signaling pathway, thereby inhibiting GC cell migration and invasion [31].

Short stature homeobox 2 (SHOX2), an oncogene, has been demonstrated to be closely related to tumor metastasis. It is a regulator of the EMT process and can promote the proliferation, migration, and invasion of malignant tumors, such as gastric and esophageal cancer [32, 33]. Research has shown that SHOX2 can regulate TβR-I in the TGF-β signaling pathway, thereby inducing the EMT process [34]. SHOX2 can also directly activate the tumor metastasis-related gene WASF3, recruit the signal transduction-related factor STAT3 at its promoter site, and form a molecular complex with STAT3, which collectively enhances the activity of WASF3, promoting tumor metastasis [35].

SRY related high mobility group box 4 (SOX4) is a crucial transcription factor that regulates tissue, organ, and nervous system growth and development. Encoded by a single-exon gene, it is one of the essential members of the SOX family C subfamily [36, 37]. Research has shown that knocking out the mouse SOX4 gene leads to defects in heart development and impaired B lymphocyte development [38]. In a total of 462 studies examining gene abnormal expression involving over 20 cancer types, 23% of them identified abnormally high expression of SOX4 in cancers such as GC, melanoma, glioblastoma, lung cancer, and breast cancer [39–42]. Silencing this gene can significantly inhibit cancer cell proliferation. SOX4 is closely associated with EMT; for example, its overexpression activates the TGF-β signaling pathway in GC, promoting the transformation of normal epithelial cells into mesenchymal cells [43]. Moreover, SOX4 plays a vital role in the crosstalk of PI3K-AKT and Wnt/β-catenin signaling pathways. The absence of this gene leads to reduced β-catenin expression, although no direct relationship exists between the two. The specific mechanism may involve the phosphorylation of AKT and subsequent downregulation of β-catenin expression, thus inhibiting tumor progression [44, 45].

PR Domain Zinc Finger Protein 1 (PRDM1) regulates B and T cell differentiation and is essential for T cell-mediated immune suppression [46]. Increasing evidence points to inconsistent expression levels of PRBM1 across various tumors, such as high expression in gastric and lung cancer and low expression in melanoma [47, 48]. Tumor cells overexpressing PRDM1 can upregulate PD-L1 expression, leading to tumor immune escape and suppression of tumor immunity. This upregulation may be related to the enhancement of USP22 transcription level by PRDM1, which decreases SPI1 protein degradation through ubiquitination and consequently increases PD-L1 expression [49].

As previous enrichment analysis results indicated that norcantharidin primarily influences BPs like cell adhesion, EMT, and cell apoptosis, this study intersected CAMs, TCGA-STAD DEGs, hallmark-EMTs, NCTDKMs, and HMGs. As a result, SERPINE1 was identified as the key target of norcantharidin intervention in GC, consistent with network pharmacology and molecular docking findings identifying SERPINE1 as one of norcantharidin’s targets. Consequently, this study conducted a comprehensive bioinformatics analysis of SERPINE1. The analysis revealed that SERPINE1 is highly expressed in GC tissue compared to normal gastric tissue and has a negative correlation with patients’ overall survival. Examining its histological staging, T, N, M staging, and other clinicopathological characteristics, a positive correlation was found between SERPINE1’s high expression and the malignant progression of gastric cancer, i.e., increased SERPINE1 expression levels correlated with faster malignant progression. Univariate and multivariate Cox regression analysis showed that SERPINE1 could serve as an independent prognostic factor for patients, with the potential to become a GC prognostic biomarker. Furthermore, SERPINE1 is involved in the immune infiltration of the GC tumor microenvironment. It is speculated that SERPINE1 expression creates an inhibitory immune microenvironment, with multiple immune cells secreting numerous anti-inflammatory cytokines, inducing tumor immune escape and accelerating tumor progression [50, 51].

## Conclusion

In this study, utilizing the constructed antitumor pCm database, we combined network pharmacology, molecular docking methods, transcriptomics, and bioinformatics analysis to identify norcantharidin as a key component in pCms and investigate its mechanism of action for GC intervention. The results suggest that norcantharidin exerts its therapeutic effect by influencing GC cells’ BPs, such as cell adhesion, migration, and inflammatory response. Our research provides evidence and a research strategy for the post-marketing reevaluation of antitumor pCms.

## Supplementary Information

**Additional file 1.** Antitumor proprietary Chinese medicines involved in this study.

**Additional file 2.** SERPINE1 expression and prognostic analysis across various cancers. A: Analysis of SERPINE1 transcription levels in the TCGA dataset; B: Analysis of SERPINE1 transcription levels in the combined TCGA + GTEx dataset; C: Survival analysis of SERPINE1 in the TCGA dataset. *p < 0.05, **p < 0.01, ***p < 0.001.

**Additional file 3.** Analysis of the association between SERPINE1 and clinical features in gastric cancer patients. A: Expression analysis of SERPINE1 across various datasets; B: Investigation of the correlation between SERPINE1 and diverse clinical features in gastric cancer patients.

**Additional file 4. **Prognostic analysis of SERPINE1 in gastric cancer patients. A: Survival analysis of SERPINE1; B: Analysis of the prognostic value of SERPINE1; C: Univariate and multivariate Cox regression analysis for the association between SERPINE1 expression and clinical features in gastric cancer patients.

## Data Availability

All the data used to support the findings of this study are available from the corresponding author upon reasonable request.

## Abbreviations

BP: Biological processes
CAMs: Cell adhesion molecule
CC: Cellular components
Ch.P: Pharmacopoeia of the People’s Republic of China 2020
EMT: Epithelial-mesenchymal transition
GC: Gastric cancer
GO: Gene Ontology
Hallmark-EMTs: EMT gene sets (EMTs) from the MSigDB Hallmark gene set
HMGs: Core molecules of the key module network
KEGG: Kyoto Encyclopedia of Genes and Genomes
MF: Molecular functions
NCTD: Norcantharidin
NCTDKMs: Targets intersected between norcantharidin and GC
NRDL: *Catalogue of Drugs for Basic National Medical Insurance/Employment Injury Insurance/Birth Insurance* (2022)
pCms: Proprietary Chinese medicines
PRDM1: PR Domain Zinc Finger Protein 1
SERPINE1: Serine protease inhibitor clade E member 1
SHOX2: Short stature homeobox 2
SOX4: SRY related high mobility group box 4
TCGA-DEGs: DEGs in patients’ normal tissues and tumor tissues from the TCGA-STAD dataset
TCM: Traditional Chinese medicine
